# Supplementary material for: Efficient and accurate spatial mixing of machine learned interatomic potentials for materials science
Source: NPJ Comput Mater. 2026 Feb 6;12(1):110. doi: 10.1038/s41524-026-01982-6 (PMC12987727; doi:10.1038/s41524-026-01982-6)
Supplement: Supplementary file 1 — Supplementary Information [file 41524_2026_1982_MOESM1_ESM.pdf]

Supplementary Information for ‘Efficient and Accurate Spatial Mixing of Machine Learned Interatomic Potentials for Materials Science’

Fraser Birks[1*], Matthew Nutter[1,2], Thomas D Swinburne[3], James R Kermode[1]

[1*]Warwick Centre for Predictive Modelling, School of Engineering, University of Warwick, Library Road, Coventry, CV4 7AL, United Kingdom.

[2]Department of Physics, University of Warwick, Library Road, Coventry, CV4 7AL, United Kingdom.

[3]Aix-Marseille Université, CNRS, CINaM UMR 7325, Campus de Luminy, Marseille, 13288, France.

*Corresponding author. E-mail: fraser.birks@warwick.ac.uk;

S1 Linear ACE potential fitting parameters

Here we present the full set of potential fitting parameters for the Fe, W and W-He ACE potentials used in the study. The main fitting parameters are presented in Table S1. Weights by config type for the Fe potential are shown in Table S2. For the W and W–He ACE, we derive energy weights for configurations following the method suggested in [1] in which the energy weight on configuration R is generated by

$$w_R = \frac{1000}{\sqrt{N_R}} \tag{1}$$

where N_R is the number of atoms in configuration R. For each configuration, the force weights were set to 10.0 and the virial weights were set to 0.0. Virials were not used in the fitting in order to avoid any errors introduced from underconverged (insufficient K-point density) DFT, as suggested by the dataset author [2]. The exception to this were the single W atom configurations (which were evaluated at a tighter K-point density), which had an energy weight of 4000.0, a force weight of 100.0 and a virial weight of 200.0.

Table S1 Fe, W and W–He ACE potential parameters. BLR stands for Bayesian linear regression.

	Fe ACE	W ACE	W–He ACE
Cutoff	5.5	5.0	5.0
Corr. order	3	3	3
Max. poly. deg.	20	21	20
Solver	BLR	BLR	BLR
Smoothness prior strength	4	4	4

Table S2 Fe ACE configuration weighting.

Config type	Energy weight	Force weight	Virial weight
default	30.0	1.0	1.0
slice_sample	80.0	0.1	1.0
prim_random	80.0	0.1	1.0

S2 Speedup and load balancing of mixed simulations

This section provides an investigation into the speedups for load balanced and non-load balanced ML-MIX simulations in silicon with a fixed expensive potential region size for (a) a fixed number of atoms and an increasing number of processors and (b) a fixed number of processors and an increasing number of atoms. It is shown that load balancing is most important when considering small domains (<30,000 atoms) and fewer MPI parallel processors (<15). It is shown that for small domains, the speedup variability between different numbers of processors is lessened by load balancing.

MD simulations were conducted with Si domains at 0 K (frozen atoms). Two sets of simulations were run, set (a) where the number of atoms were fixed and the number of processors were increased between 1 and 48, and set (b) where the number of processors was fixed and the number of atoms were increased between 8×10^3 and 10^6. For each separate simulation domain, timing measurements were carried out with (i) only the 2_10 ACE Si cheap potential, (ii) only the 4_20 ACE Si expensive potential, (iii) load-balanced and (iv) non-load balanced ML/ML simulations containing both the expensive and cheap potentials. Within each simulation domain, the same number of timesteps was used for each timing measurement (i-iv), with the number selected such that the wall-time of the longest (the all-expensive) simulation was approximately 45 minutes. To build the expensive domain in each ML/ML simulation, single seed atoms were selected at the center of each cell, around which 6 Å core regions and 6 Å buffer regions were constructed. This meant that the number of expensive potential atoms was fixed across all ML/ML simulations, regardless of overall domain size.

For set (a), two atomistic domain sizes were chosen; a 'small' domain of 8000 atoms, and a 'large' domain of 262,144 atoms. For set (b), the number of processors was set at 27 for all domain sizes used. For each simulation domain investigated, the 'upper-limit' speedup S_{UL} was computed with

$$S_{\mathrm{UL}} = \frac{N S_{\mathrm{C/E}}}{N_{\mathrm{E}} S_{\mathrm{C/E}} + N_{\mathrm{C}}}, \tag{2}$$

where N is the total number of atoms in a simulation domain, N_{E} and N_{C} are the number of expensive and cheap atoms (including those in both buffer regions), and $S_{\mathrm{C/E}}$ is the measured speedup of the all-cheap simulation over the all-expensive simulation in the domain of interest.

Simulations were carried out on Dell PowerEdge C6420 compute nodes each with 2 x Intel Xeon Platinum 826 (Cascade Lake) 2.9 GHz 24-core processors; 48 cores per node; 192 GB DDR4-2933 RAM per node; 4 GB RAM per core.

S2.1 Load balancing strategies

Load-balancing is performed via the `fix balance` command in `LAMMPS`. If the `time` keyword is specified `LAMMPS` attempts to dynamically decompose the overall domain into subdomains of equal computational cost. `LAMMPS` has two strategies to decompose domains - 'brick' and 'tiled'. With the 'brick' strategy, each individual sub-domain is cuboidal, and is constrained to be joined at the corners. With the 'tiled' strategy, each domain is also cuboidal, but not necessarily constrained to connect at corners. For more details, please refer to the `comm_style` page of the `LAMMPS` documentation [3]. Within the `fix balance` command, two algorithms are available; the `shift` algorithm, compatible with both the 'brick' and 'tiled' decomposition strategies, and the `rcb` algorithm, compatible with just the 'tiled' decomposition strategy. For more detail on these algorithms, please refer to the `fix balance` page of the `LAMMPS` documentation [4]. Each load

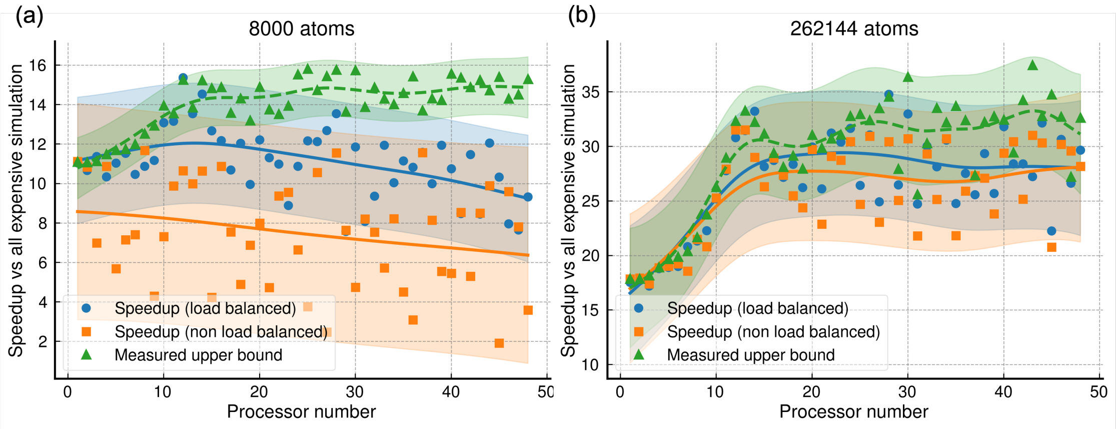

Fig. S1 Speedup of ML/ML simulations over all-expensive reference simulations on an increasing number of processors for two domain sizes, both with load-balancing (blue points) and without (orange points). In each case, the upper bound speedup (green line) is determined from equation 2. (a): Speedup on a small (8000 atom) domain. For this domain size, one can only attain near-upper bound speedups for small numbers of processors if a load-balancing strategy is used. The effect of load balancing is very pronounced, as without it all expensive atoms frequently end up on one sub-domain, creating simulation bottle-necks. (b): Speedup on a large (262,144 atom) domain. At this domain size, one attains a near upper-bound speedup for any number of MPI parallel domains, and load balancing has a relatively minor effect. All trend-lines are fitted with Gaussian process regression.

balancing strategy was investigated, and it was found for a simple domain with one central expensive region, the `shift` algorithm performed marginally better when domains were small and the `rcb` algorithm performed marginally better when domains were large. Throughout this paper, the `shift` algorithm is used with the brick domain decomposition strategy. We recommend users test each load-balancing strategy to determine which works best for their system.

S2.2 Results and discussion

S2.2.1 Fixed atoms, increasing processors

The results for simulation set (a) are displayed in Fig. S1. For the smaller (8000 atom) domain, it can be seen that with load balancing, a near-upper-bound speedup can be attained for a number of processors below ~ 15. Above this, the load balanced simulation falls significantly below the upper-bound due to communication overheads taking up a significant fraction of the runtime. Without load balancing, the performance is on average significantly worse, but varies greatly between the number of processors chosen. Particularly poor performances are due to all of the expensive atoms landing on a single parallel domain. For the larger (262,144 atom) domain, it can be seen that near-upper bound speedup is approximately attained for any processor number, regardless of load-balancing. This arises due to every individual subdomain always being far larger than the expensive potential region.

S2.2.2 Fixed processors, increasing atoms

Results for simulation set (b) in which the simulation domain size is increased at a fixed number of processors (27) is shown in Fig. S2. For smaller atomic domains (<30,000 atoms), it can be seen that load balancing is important, providing a considerable speedup. As the size of the domain gets larger, load-balancing becomes less important, and the overall speedup tends toward the overhead-free upper limit.

S3 Impact of constrained fitting

In this paper, the cheap potentials have been constrained to enforce elastic constant matching. In this section, we show that matching the elastic constants between our potentials leads to reduced errors in ML-MIX relaxed structures for less than 3% strain deviations from the bulk.

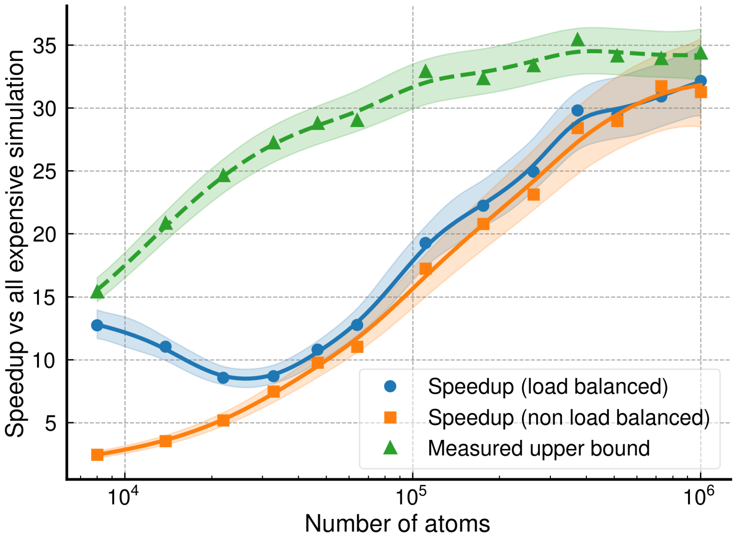

Fig. S2 The measured speedup of the mixed simulation over the all expensive simulation on 27 processors, for increasing numbers of atoms both with load-balancing (blue points) and without (orange points). The green line indicates the maximum possible speedup (with no overheads), computed from equation 2. It can be seen that for small numbers of atoms (<30,000) load balancing is important, and for large number of atoms ($\sim 10^6$) the speedup tends toward the upper bound. Trend lines have been fitted with Gaussian process regression.

A periodic $10 \times 10 \times 10$ (8000 atom) bulk Si structure was constructed and sheared in the xy to directions to strains between 0 and 7%. For each structure, a joint ML/ML relaxation was performed using ML-MIX. The central atom and the surrounding 6 Å were modeled expensively, with the remainder of the atoms were with the constrained or unconstrained Si 2_10 cheap potentials in two separate investigations. For the relaxation, a blending region of 6.0 Å was used, as well as a relatively loose tolerance of $\|\mathbf{F}\|_2 < 10^{-3}$ eV/Å. Smaller tolerances than this would frequently fail to converge. The strain error Du was then found for each structure as

$$Du = \|\nabla u - \nabla u_{\mathrm{ref}}\|_2 \tag{3}$$

where u is the displacement with respect to the ML/ML relaxed structure and u_{ref} is the displacement field from the relaxed all-expensive reference.

S3.1 Results and discussion

Fig. S3 shows the strain error in the ML/ML relaxed structures. The spatial distribution of the error in the strain fields matches that expected from an Eshelby inclusion, and it can be seen that for very small strains $\epsilon_{xy} < 0.005$, the constrained potential exhibits very little error in the strain whilst the constrained potential error increases immediately. The constrained potential continues to have lower strain error up until $\epsilon_{xy} = 0.03$.

S4 Energy conservation

A fundamental limitation of force mixing is a lack of energy conservation. Whilst this has been noted previously [5], the features in a force-mixed simulation that may worsen or improve energy conservation are unclear. Here we present a brief investigation where we test the impact on energy conservation of (i) having a larger interface region between potentials, (ii) having a more flexible cheap potential and (iii) introducing a larger blending region between potentials. Whilst findings presented here are derived from ML/ML simulations using ML-MIX, the findings are applicable to force-mixing simulations more generally, including QM/MM simulations.

A periodic cube of Si was constructed which matches that described in section 2.3 of the main manuscript. The spherical central region of the domain (around the fixed stretched bond) was simulated expensively, with the remainder of the domain modeled cheaply. The expensive potential was the same Si

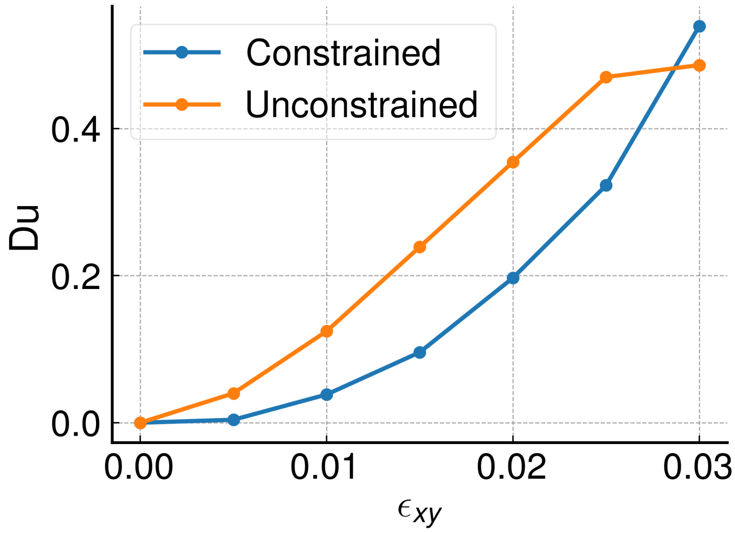

Fig. S3 The strain error norm Du measured for sheared and relaxed bulk Si ML/ML simulations using cheap potentials which are a both constrained (blue points) and unconstrained (orange points) for different applied shear strain. The constrained cheap potential has elastic constants that closely match the expensive potential. The ML/ML simulation contains a central 6 Å sphere of the expensive potential surrounded by the cheap potential. *Du* is far greater for small strains when using the unconstrained cheap potential, as the mismatched elastic constants to the expensive lead to Eshelby-inclusion type strain fields.

Table S3 Si cheap potential parameters.

	2_10	2_15	3_15
Cutoff	6.0	6.0	6.0
Corr. order	2	2	3
Max. poly. deg.	10	15	15

ACE used in section 2.3 of the main manuscript. Three different cheap ACE potentials were used, each fit according to the constrained fitting procedure outlined in the main manuscript. The key parameters of these potentials are displayed in Table S3. To investigate energy drift in the simulation set-up, a 50 ps, 300 K ML-MIX `LAMMPS` MD simulation was performed with a timestep of 1 fs. As the total energy of a force-mixed simulation is not defined, the total energy of the full system was measured through evaluating snapshots output every ps with the expensive potential.

Two separate investigations were conducted for each cheap potential. (a) For a set-up with no blending region (abrupt force-mixing) the size of the spherical expensive potential region was varied between 4 and 10 Å, and (b) for a fixed 10 Å core + blending region, the amount of blending region is varied between 4 and 6 Å. Both linear and cubic blending were investigated.

S4.1 Results and discussion

S4.1.1 Size of potential-potential interface

The energy over time for the abruptly force-mixed ML/ML simulations are shown on the left of Fig. S4. Two facts are discernible from these plots: the energy drifts are approximately linear, and larger expensive potential regions lead to greater energy gain. The right hand side of Fig. S4 shows the energy gains normalized by the (approximately spherical) surface interface area between potentials. Under this normalization, the curves collapse onto a single straight line, giving us the result that for two given potentials and a given blending regime the rate of energy increase per unit surface contact area is approximately constant. The gradient of this line can be thought of as the energy flux from the force mixing contact surface into the material.

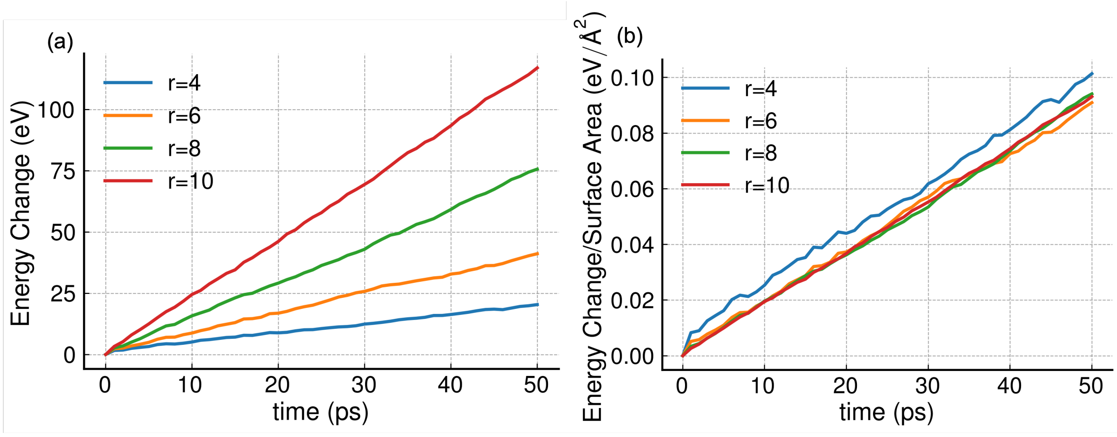

Fig. S4 Energy drift over time for an abruptly mixed ML/ML simulation of bulk Si, using the 2_10 cheap potential. The energies are that of the full system, computed at snapshots every ps with the expensive potential. Blue, orange, green and red curves refer to sizes of the expensive potential region of 4, 6, 8 and 10 Å respectively. (a): The raw energy change for each simulation, showing that a larger expensive potential region causes a greater rate of energy increase. (b): Energy change normalized by spherical interfacial contact area between potentials, showing that for a given two potentials in abrupt contact the rate of energy change per unit of contact area (energy flux) is constant.

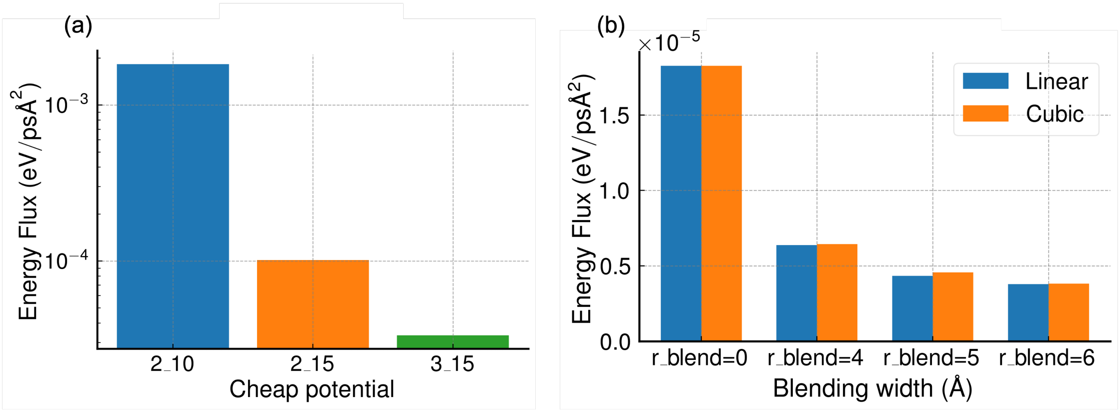

Fig. S5 Measurements of (positive) energy flux from the ML/ML boundary for different cheap potentials and blending regimes. (a): Energy flux for abruptly mixed ML/ML simulations with different cheap potentials of increasing complexity. Blue, orange and green bars represent the measured flux for the 2_10, 2_15 and 3_15 cheap potentials respectively. More complex cheap potentials lead to orders of magnitude lower energy drift. (b): Energy flux for ML/ML simulations with the 2_10 potential with different blending widths and blending functions. r_blend=0 corresponds to abrupt mixing, whilst the other bars correspond to increasing amounts of blending. Increasing the blending width decreases the energy flux from the potential contact surface. Very little difference in energy flux was observed between using a linear blending function (blue bars) or cubic blending function (orange bars).

S4.1.2 Flexibility of cheap potential

Energy fluxes from ML/ML simulations of three different cheap potentials are shown on the left-hand side of Fig. S5. Note that as the cheap potential complexity increases, the energy flux from the internal potential/potential contact surface decreases by orders of magnitude. The rationale for this is straightforward; more complex cheap potentials with greater numbers of basis functions have the flexibility to better approximate the local potential energy surface of the expensive potential.

S4.1.3 Width of blending region

The right-hand side of Fig. S5 shows how adding a blending region in the ML-MIX simulation can decrease the energy flux. The left-most bars correspond to abrupt mixing, and it can be seen that as core region is swapped out for blending region the amount of energy drift in the simulation decreases. The type of blending (linear or cubic) did not make any noticeable difference to measured energy flux.

References

[1] Witt W. C. et al. "Acepotentials.jl: a julia implementation of the atomic cluster expansion". In: *The journal of chemical physics* **159**.16 (Oct. 2023), p. 164101. ISSN: 0021-9606. DOI: 10.1063/5.0158783.

[2] Nutter M., Kermode J. & Bartók-Pártay A. *Supplementary Material for: "Kink-Helium Interactions in Tungsten: Opposing Effects of Assisted Nucleation and Hindered Migration"*. eng. June 2024. DOI: 10.5281/zenodo.11620484. URL: https://zenodo.org/records/11620484.

[3] Berger R. & Kohlmeyer A. *Comm_style command*. Accessed: 2025-02-06. 2019. URL: https://docs.lammps.org/comm_style.html.

[4] Berger R., Kohlmeyer A., Plimpton S. & Gissinger J. *Fix balance command*. Accessed: 2025-02-06. 2019. URL: https://docs.lammps.org/fix_balance.html.

[5] Bernstein N., Kermode J. R. & Csányi G. "Hybrid atomistic simulation methods for materials systems". en. In: *Reports on progress in physics* **72**.2 (Jan. 2009), p. 026501. ISSN: 0034-4885. DOI: 10.1088/0034-4885/72/2/026501.
